# Effects of PARP-1 Deficiency and Histamine H_4_ Receptor Inhibition in an Inflammatory Model of Lung Fibrosis in Mice

**DOI:** 10.3389/fphar.2019.00525

**Published:** 2019-05-16

**Authors:** Mariaconcetta Durante, Silvia Sgambellone, Cecilia Lanzi, Patrizia Nardini, Alessandro Pini, Flavio Moroni, Emanuela Masini, Laura Lucarini

**Affiliations:** ^1^ Section of Pharmacology, Department of Neurosciences, Psychology, Drug Research and Child Health (NEUROFARBA), University of Florence, Florence, Italy; ^2^ Medical Toxicology Unit, Careggi-University Hospital (AOUC), Florence, Italy; ^3^ Section of Histology, Department of Clinical and Experimental Medicine, University of Florence, Florence, Italy

**Keywords:** histamine H_4_ receptors, poly(ADP-ribose) polymerase 1, pulmonary fibrosis, inflammation, oxidative stress, cytokines

## Abstract

Pulmonary fibrosis is the most frequent form of interstitial lung disease. Effective therapies are not yet available; novel therapeutic approaches are needed for counteracting fibrosis. Poly(ADP-ribose) polymerases are enzymes, involved in DNA repair and cell apoptosis. PARP-1 deficient mice exhibited reduced lung fibrosis in response to bleomycin treatment compared to wild-type controls. Histamine H_4_ receptors (H_4_Rs) have been recognized as a new target for inflammatory and immune diseases, and H_4_R ligands reduced inflammation and oxidative stress in lung tissue. The aim of the study was to evaluate the cross-talk between PARP-1 and H_4_R in a model of bleomycin-induced lung fibrosis in PARP-1^−/−^ and WT mice. Animals were treated with bleomycin or saline by intra-tracheal injection. JNJ7777120, an H_4_R antagonist, or VUF8430, an H_4_R agonist, were administered i.p for 21 days. Airway resistance to inflation was evaluated, and lung tissues were processed for PARylated protein content, oxidative stress evaluation, and histology of small bronchi. The levels of pro-inflammatory (IL-1β and TNF-α), regulatory (IL-10), and pro-fibrotic (TGF-β) cytokines were evaluated. The deposition of αSMA was determined by immunofluorescence analysis. The results indicate that JNJ7777120 reduces PARylated protein production, decreases oxidative stress damage, and MPO, a marker for leukocyte tissue infiltration, in PARP-1^−/−^ mice. A significant decrease in the production of both IL-1β and TNF-α and a significant increase in IL-10 levels are observed in mice treated with H_4_R antagonist, suggesting a crucial anti-inflammatory activity of JNJ7777120. The smooth muscle layer thickness, the goblet cell relative number, and collagen deposition decreased following JNJ7777120 administration. The H_4_R antagonist treatment also reduces TGF-β production and αSMA deposition, suggesting an important role of JNJ7777120 in airway remodeling. Our results show that PARylation is essential for the pathogenesis of pulmonary fibrosis and propose that PARP-1 and H_4_Rs are both involved in inflammatory and fibrotic responses. JNJ7777120 treatment, in a condition of PARP-1 inhibition, exerts anti-inflammatory and anti-fibrotic effects, reducing airway remodeling and bronchoconstriction. Therefore, selective inhibition of H_4_Rs together with non-toxic doses of selective PARP-1 inhibitors could have clinical relevance for the treatment of idiopathic pulmonary fibrosis.

## Introduction

Idiopathic pulmonary fibrosis (IPF) is a severe and progressive lung disease with approximately 3 years of median survival from the time of diagnosis and great associated morbidity, with wide-ranging negative effects on quality of life (Swigris et al., 2005). Despite recent advances in understanding the disease pathobiology, IPF remains a disease with a poor prognosis and an incompletely understood pathogenesis. In the last few years, the European Medicines Agency and the Food and Drug Administration (FDA) approved the use of nintedanib and pirfenidone as new safe drugs for IPF treatment. However, effective treatments are not available, and new therapeutic strategies are needed as alternative options when the standards of care are not sufficient or effective (Wynn, 2007; Paz and Shoenfeld, 2010).

Despite an unknown etiology, many studies identified a significant genetic risk associated with the development of IPF, such as SFTPC, MUC5B, and telomerase mutations; however, mechanisms by which genetic risk factors promoting IPF remain unclear (Allen et al., 2017). It has been proposed that pulmonary fibrosis is due to an augmented proliferation of fibroblasts with massive formation and accumulation of extracellular matrix (ECM) proteins (e.g., vimentin, collagen, and fibronectin) that eventually damage lung function (Wilson and Wynn, 2009). Deposition of “scar” tissue in the *interstitium* of the lung causes an alteration in the homeostatic cross-talk between epithelial and mesenchymal cells. Epithelial cells secrete anti-fibrotic mediators like prostaglandin E_2_ (PGE_2_) (Lama et al., 2002); thus, the loss of epithelial cells results in lower levels of PGE_2_, which in turn, can allow resident fibroblasts to proliferate and differentiate into alpha-smooth muscle actin (αSMA) positive myofibroblasts (Kolodsick et al., 2003). Additionally, the release of the transforming growth factor-β (TGF-β), the most potent pro-fibrotic growth factor, promotes apoptosis of epithelial cells while simultaneously prevents apoptosis in lung fibroblasts (Thannickal and Horowitz, 2006). The apoptosis paradox allows resident fibroblasts to accumulate and become myofibroblasts. Myofibroblasts, organized into agglomerations of cells known as fibroblastic foci, are highly secretory cells producing an excessive tissue matrix, especially collagen, and highly contractile cells causing distortion of the alveolar architecture. When the synthesis of new collagen by myofibroblasts overcomes its degradation rate, pulmonary fibrosis occurs leading to the accumulation of collagen (Wynn, 2008), the common pathological hallmark of fibrotic disorders. This process results in multiple alterations in the lung structure, with progressive thickening of the air-blood membrane and airway stiffening; these lesions impair both gas diffusion and ventilation/perfusion relationship, with reduction or loss of gas exchange capacity (Plantier et al., 2018).

Poly(ADP-ribose) polymerases (PARPs) are enzymes, involved in DNA repair and apoptosis. PARP-1 is the most abundant member of the PARP family and the most widely studied enzyme of this class. PARP-1 is activated upon binding to single- and double-strand DNA breaks *via* its N-terminal zinc finger domains (Ali et al., 2012; Langelier et al., 2012). Once activated by DNA damage, PARP-1 widely poly(ADP-ribosyl)ates itself and promotes the enrollment of DNA repair proteins that are required for lesion processing and repair. However, when DNA damage is severe, PARP-1 becomes over-activated leading to excessive consumption of NAD^+^ and consequently to depletion of ATP that results in cellular dysfunction and necrotic cell death.

It has been reported that PARP activation characterizes a key pathway in many pathophysiological conditions associated with inflammation and oxidative stress. Interestingly, genes targeting approaches and the use of non-selective inhibitors have shown that PARP-1 is involved in a number of fibrotic diseases affecting the heart (Pacher et al., 2002), liver (Mukhopadhyay et al., 2014), vessels (Abdallah et al., 2007), and lungs (Genovese et al., 2005). Moreover, recent studies demonstrated that genetic depletion and pharmacological inhibition of PARP-1 reduced pulmonary fibrosis in an animal model of bleomycin-induced lung injury (Hu et al., 2013; Lucarini et al., 2017), suggesting that PARylation is important for myofibroblast differentiation and for the pathogenesis of the disease.

Histamine H_4_ receptor (H_4_R) has been identified as a novel target for inflammatory and immune disorders (Gschwandtner et al., 2013). Recent studies demonstrated that H_4_Rs are involved in the prevention of fibronectin-induced lung fibroblast migration, suggesting these receptors as new targets for the treatment of lung fibrosis (Kohyama et al., 2010). Moreover, H_4_R antagonists significantly reduce goblet cell hyperplasia and collagen deposition in a model of allergic asthma (Masini et al., 2013) and decrease inflammation and oxidative stress in a bleomycin-induced lung injury model (Rosa et al., 2014; Lucarini et al., 2017).

However, the cellular mechanism underlying the beneficial effects of H_4_R antagonists in lung diseases are not completely understood, and this topic needs to be investigated in details.

The aim of the present study is to evaluate a possible involvement of PARP-1 in the H_4_R antagonists’ beneficial effects in reducing airway inflammation and remodeling. For this purpose, we evaluated the effects of the H_4_R-selective antagonist, JNJ7777120, and of the H_4_R agonist, VUF8430, in an *in vivo* model of bleomycin-induced lung fibrosis, in PARP-1 *knock-out* and in *wild-type* mice.

## Materials and Methods

### Drugs and Reagents

Compound JNJ7777120 (JNJ; 1-[(5-chloro-1H-indol-2-yl)carbonyl]-4-methylpiperazine), a potent and selective H_4_R antagonist, has been used at a concentration of 2.5 mg/kg of body weight (b.wt.). This compound was kindly provided by Dr. Robin Thurmond (Janssen Research & Development, USA). Compound VUF8430, (VUF; 2-[(aminoiminomethyl)amino] ethyl carbamodithioic acid ester, S-(2-guanidylethyl)-isothiourea), an H_4_R agonist, (Sigma-Aldrich, San Louis, MO, USA), was used at a concentration of 2 mg/kg b.wt. Bleomycin (Merck-Millipore, Burlington, MA, USA) was used at a concentration of 0.05 IU for each mouse and dissolved in 50 μl of saline, in order to obtain the bleomycin-induced model of lung fibrosis. The drug doses and frequency of administrations were selected according to previous papers (Rosa et al., 2014; Lucarini et al., 2016).

### Animals

Male wild-type (WT) C57BL/6 mice, approximately weighing 25–30 g and 8–9 weeks old, purchased from a commercial dealer (Harlan, Udine, Italy), and male C57BL/6 PARP-1 deficient mice (PARP-1^−/−^) (Wang et al., 1995), comparable in mice strain, age, and weight with WT mice, were used for the experiments. All the animals were fed with standard diet and water at libitum and housed under a 12 h light/dark photoperiod at 22°C for at least 48 h before the experiments.

The study protocol complied with the recommendations of the European Economic Community (86/609/CEE) and the Declaration of Helsinki on animal experimentation and was approved by the animal Ethical and Care Committee of the University of Florence (Florence, Italy) and by the Italian Health Ministry (Authorization n 874/2017-PR). Experiments were performed at the Centre for Laboratory Animal Housing and Experimentation (CeSAL) at the University of Florence. The ARRIVE guidelines were considered (McGrath et al., 2010).

### Surgery and Treatments

Seventy mice (30 WT and 40 PARP-1^−/−^) were anesthetized with zolazepam/tiletamine (Zoletil, 50/50 mg/ml, Virbac Srl, Milan, Italy; 50 μg/g, in 100 μl of saline, i.p.); 50 of them (20 WT and 30 PARP-1^−/−^) were intra-tracheally treated with bleomycin (0.05 IU in 50 μl of saline), and the other 20 (10 WT and 10 PARP-1^−/−^) were intra-tracheally treated with 50 μl of saline (referred to as non-fibrotic negative controls, Naive).

Ten bleomycin-treated WT and 10 bleomycin-treated PARP-1 deficient mice received two daily intra-peritoneal (i.p.) injections of 100 μl of JNJ solution (2.5 mg/kg; i.p.), after bleomycin administration and for the next 21 days. These are referred to as Bleomycin+JNJ treated groups. Ten bleomycin treated PARP-1^−/−^ mice received two daily intra-peritoneal injection of 100 μl of VUF solution (2 mg/kg; i.p.), after bleomycin administration and for the next 21 days. These are referred to as Bleomycin+VUF treated group. Ten WT mice and 10 PARP-1^−/−^ mice were treated only with vehicle (PBS) and referred to as fibrotic positive controls (Bleomycin+Vehicle).

### Functional Assay of Fibrosis

At the end of the treatment period, all mice were subjected to the measurement of airway resistance to inflation and static lung compliance, functional parameters for fibrosis-induced lung stiffness. The measurement is performed using a constant volume mechanical ventilation method with constant number of breaths in a minute; the static compliance determination is performed applying a positive end-expiratory pressure of 3 cm H_2_O, to mimic spontaneous ventilation (Pini et al., 2010, 2012; Manni et al., 2016). Briefly, a 22-gauge cannula (Venflon 2; Viggo Spectramed, Windlesham, UK, 0.8 mm diameter) was inserted into the trachea of the anesthetized mouse. The animal was ventilated with a small-animal respirator (Ugo Basile, Comerio, Italy) adjusted to deliver a tidal volume of 0.8 ml at a rate of 20 strokes/min. A high-sensitivity pressure transducer (settings: gain 1, chart speed 25 mm/sec.; P75 type 379; Harvard Apparatus Inc., Holliston, MA, USA) connected to a polygraph (Harvard Apparatus Inc. Edenbridge, UK; settings: gain 1, chart speed 25 mm/s) was used to register the changes in lung resistance to inflation, defined as the pressure at airway opening (PAO). Changes in lung resistance to inflation, expressed as millimeters on the chart and registered for at least 3 min, were carried out not less than 40 consecutive tracings of respiratory strokes and then averaged. For static lung compliance determination, multiple linear regression was used to fit pressure and volume in each individual mouse to the linear model of the lung (Manni et al., 2016).

### Lung Tissue Sampling

After PAO measurements, the animals were killed with lethal dose of anesthetic drugs. The whole left lungs were removed and fixed with 4% paraformaldehyde in PBS for histological analysis. The right lungs were weighed, quickly frozen, and stored at −80°C. For biochemical measurements, the samples were thawed at 4°C, homogenized on ice in 50 mM Tris-HCl buffer (180 mM KCl and 10 mM EDTA, pH 7.4), and then centrifuged for 30 min at 10,000 g at 4°C, unless otherwise reported. The homogenized supernatants were collected.

### Histology and Assessment of Collagen Deposition, Goblet Cell Hyperplasia, and Smooth Muscle Layer Thickness

Six μm thick histological sections were cut from the paraffin-embedded lung samples. In order to minimize artefactual differences in the staining process, all sections were stained in a single session. A digital camera connected to a light microscope equipped with an ×20 objective was used to randomly take photomicrographs of the histological slides. Computer-aided densitometry was performed to obtain quantitative assessment of the stained sections. The free-share ImageJ 1.33 image analysis program^1^ was used to measure optical density (OD) and surface area. For each measured parameter, values are means ± SEM of the OD measurements (arbitrary units) of individual mouse (five images each) from every experimental groups (tested blind).

The assessment of lung collagen was obtained by staining the histological sections with a simplified Azan method for collagen fibers (Smolle et al., 1996), omitting azocarminium and orange G to reduce parenchymal tissue background. OD measurements of the aniline blue-stained collagen fibers were performed after selection of a correct threshold to eliminate aerial air spaces and bronchial/alveolar epithelium (Formigli et al., 2007). To confirm the assessment of lung collagen, the Picrosirius red staining was carried out; the sections were stained in 0.1% Direct Red 80/Sirius Red F3B (Sigma-Aldrich) in saturated picric acid at room temperature for 1 h, and then they were differentiated in 0.5% acetic acid, prior to dehydration, clearing, and mounting (Lattouf et al., 2014; Marcos-Garcés et al., 2017).

Moreover, lung tissue sections were stained with hematoxylin and eosin or with periodic acid-Schiff (PAS) staining for mucins in order to obtain morphometry of smooth muscle layer thickness and bronchial goblet cell number, respectively, both key markers of airway remodeling. Digital microphotographs of small-sized bronchi were taken randomly. The thickness of the bronchial smooth muscle layer was measured on the digitized images using the above-mentioned software. Total bronchial epithelial cells and PAS-stained goblet cells were counted on bronchial cross-section profiles, and the percentage of goblet cells was calculated.

### Western Blot Analysis of PARylated Protein Content

For determination of PARylated protein content in the lung, tissues were homogenized in RIPA buffer plus a cocktail of protease inhibitors and centrifuged at 12,000 g for 5 min. Supernatants were collected, and total protein levels were measured using Micro BCA Protein Assay (ThermoFisher Scientific, Waltham, MA, USA). Thirty μg of proteins were used for Western blot analysis, with a mouse monoclonal anti-PAR(10H) antibody (Alexis Biochemicals, Florence, Italy) diluted 1:1,000 in PBS-T containing 5% non-fat dry milk. The PAR(10H) monoclonal antibody recognizes poly(ADP-ribose) synthesized by PARP enzymes. A suitable peroxidase-conjugated secondary antibody (diluted 1:5,000 in PBS-T containing 5% non-fat dry milk) was used to determine the binding of the primary antibody. The loading transfer of equal amounts of proteins was controlled by reblotting the membrane with an anti-β-actin antibody (diluted 1:20,000 in 5% non-fat dry milk-PBS-T, Sigma-Aldrich). Bands were visualized by enhanced chemiluminescence (Luminata Crescendo Western HRP substrate, Merck Millipore) and quantified by densitometric analysis with the ImageJ software.

### Determination of Transforming Growth Factor-β (TGF-β), Interleukin-1β (IL-1β), Tumor Necrosis Factor-α (TNF-α), and Interleukin-10 (IL-10)

The levels of TGF-β, the main profibrotic cytokine involved in fibroblast activation, and two pro-inflammatory cytokines, IL-1β and TNF-α, were measured on aliquots (20 μl) of lung samples homogenated in PBS by using the FlowCytomix assay (Bender Medsystems GmbH, Vienna, Austria), following the protocol provided by the manufacturer. Briefly, suspensions of anti-TGF-β, IL-1β or TNF-α-coated beads were incubated with the supernatant samples and then with biotin-conjugated secondary antibodies and streptavidin-phycoerythrin. Fluorescence was read with a cytofluorimeter (CyFlow® Space, Partec, Carate Brianza, MB, Italy).

The levels of the anti-inflammatory cytokine IL-10 were measured on aliquots (100 μl) of lung homogenate supernatants by using the mouse IL-10 ELISA Ready-SET&Go!® assay (eBioscience, San Diego, USA), following the protocol provided by the manufacturer. Values are indicated as means ± SEM of 10 individual mice from each group and expressed as pg/μg of total proteins determined over an albumin standard curve.

### Determination of αSMA Deposition

Immunofluorescence analysis was performed as previously described (Lucarini et al., 2014). Briefly, histological sections of 5 μm thick were deparaffinized and boiled for 10 min in sodium citrate buffer (10 mM, pH 6.0, Bio-Optica, Milan, Italy) for antigen retrieval. A pre-incubation in 1.5% bovine serum albumin (BSA) in PBS, pH 7.4 for 20 min at RT was necessary to minimize the unspecific binding; whereupon, the sections were incubated overnight at 4°C with rabbit monoclonal anti-αSMA antibody (1:200 ABCAM, USA) followed by goat anti-rabbit Alexa Fluor 488-conjugated IgG (1:300 Invitrogen, San Diego, CA, USA) for 2 h in the dark at RT. Negative controls were performed with non-immune rabbit serum substituted for the primary antibody. The counterstaining of nuclei was obtained with 4′,6-diamidino-2-phenylindole (DAPI). Representative images were acquired with an Olympus BX63 microscope coupled to CellSens Dimension Imaging Software version 1.6 (Olympus, Milan, Italy). αSMA expression was quantified by densitometric analysis of fluorescence signal intensity, measured on digitized images using ImageJ software^2^. Twenty regions of interest (ROI) were evaluated for each sample. Values are expressed as mean ± SEM of the OD measurements (arbitrary units) of individual mouse from the different experimental groups.

### Determination of 8-Hydroxy-Deoxyguanosine (8-OHdG)

Lung DNA isolation was performed as previously described (Lodovici et al., 2000) with minor modifications. In brief, lung samples were homogenized in 1 ml of 10 mM PBS, pH 7.4, sonicated on ice for 1 min, added to 1 ml of 10 mM Tris-HCl buffer, pH 8, containing 10 mM EDTA, 10 mM NaCl, and 0.5% SDS, and incubated for 1 h at 37°C with 20 μg/ml RNase 1 (Sigma-Aldrich). Samples were incubated at 37°C overnight in the presence of 100 μg/ml proteinase K (Sigma-Aldrich). The mixture was extracted with chloroform/isoamyl alcohol (10:2, v/v). DNA was precipitated from the aqueous phase with 0.2 volumes of 10 M ammonium acetate, solubilized in 200 μl of 20 mM acetate buffer, pH 5.3, and denatured at 90°C for 3 min. The extract was supplemented with 10 IU of P1 nuclease (Sigma-Aldrich) in 10 μl and incubated for 1 h at 37°C with 5 IU of alkaline phosphatase (Sigma-Aldrich) in 0.4 M phosphate buffer, pH 8.8. All the procedures were performed in the dark. The mixture was filtered by an Amicon Micropure-EZ filter (Merck-Millipore), and 100 μl of each sample were used for 8-OHdG determination by using an ELISA kit (JalCA, Shizuoka, Japan), following the instructions provided by the manufacturer. The absorbance of the chromogenic product was measured at 450 nm. The results were calculated from a standard curve based on an 8-OHdG solution and expressed as ng of 8-OHdG/ng of total DNA.

### Determination of Myeloperoxidase Activity (MPO)

Frozen lung samples were weighed and homogenized (10 μl/mg of tissue) in 0.2 M phosphate buffer solution (PBS), pH 6, supplemented with protease inhibitors (1 mM PMSF, 20 μg/ml leupeptin, 1 μg/ml pepstatin, 1 mg/ml Pefabloc SC, and 2.5 μg/ml aprotinin, Sigma-Aldrich) and were centrifuged at 10,000 g at 4°C for 30 min. MPO was measured in the supernatants with a specific immunoassay kit (CardioMPO; PrognostiX, Cleveland, OH), according to the manufacturer’s instructions (Pini et al., 2010). Total protein concentration in the lung tissue samples was determined over an albumin standard curve. The results are expressed as picomoles/mg of protein. Values are means ± SEM of individual mice from different experimental groups.

### Statistical Analysis

Data were reported as mean values (±SEM) of individual average measures of the different animals per group, for each assay. Significance of differences among the groups was evaluated by one-way ANOVA followed by Newman-Keuls *post hoc* test for multiple comparisons. Calculations were made with Prism 5 statistical software (GraphPad Software, Inc., USA). A probability value *p* < 0.05 was considered significant.

## Results

### Functional Assay of Fibrosis (PAO)

**Figure 1:**
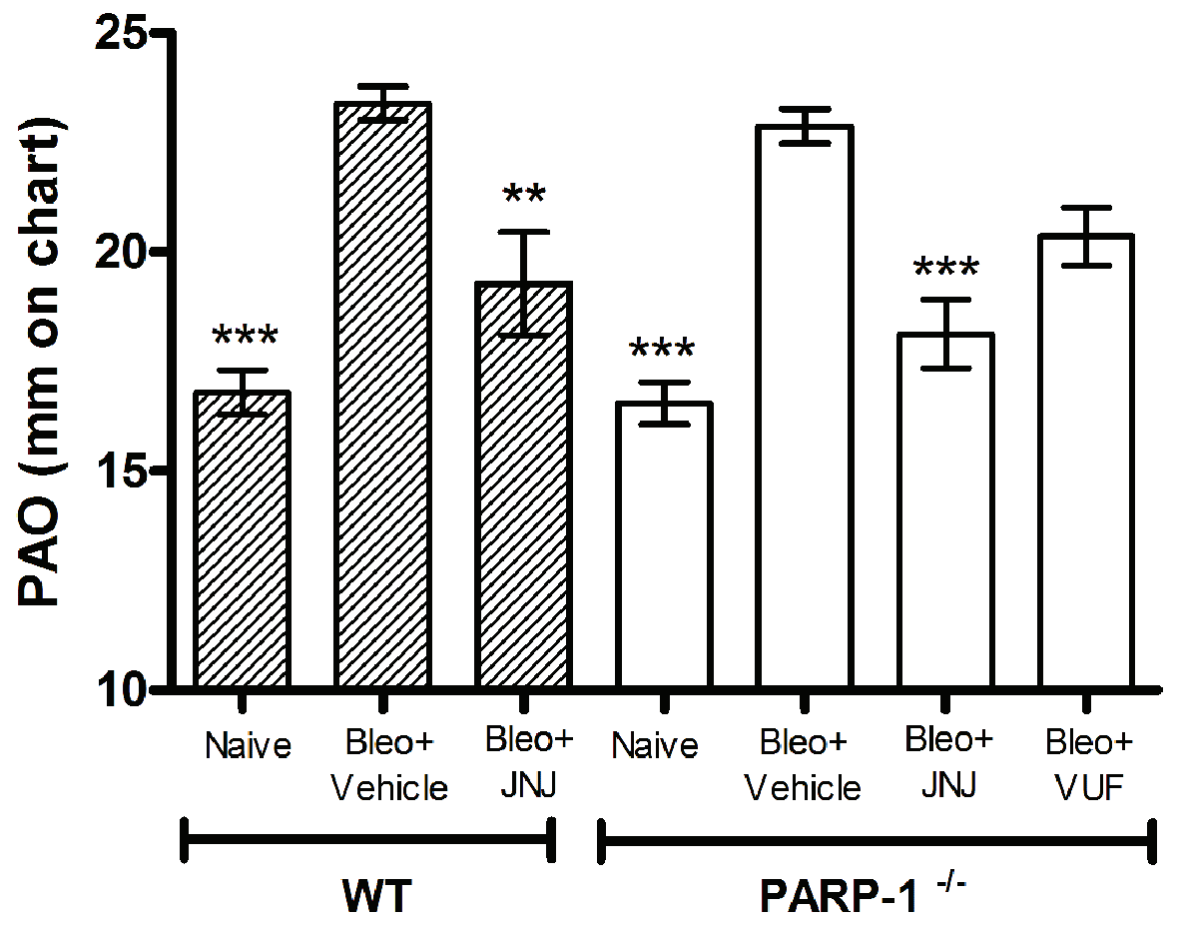
Spirometric evaluation. Bar graph and statistical analysis of differences in PAO values (means ± SEM) between different experimental groups (*n* = 10 animals per group). **p < 0.01 and ***p < 0.001 *vs.* Bleo+Vehicle of each related group WT or PARP^−/−^ (Bleo = Bleomycin).

Intra-tracheal administration of bleomycin causes an increase in airway stiffness leading to a clear-cut elevation of the pressure in airway opening (PAO) (Masini et al., 2005). Intra-tracheal delivery of bleomycin to rodents results in an intense inflammatory reaction within the first week, followed by the development of fibrosis by day 14, with maximal responses at day 21. Therefore, bleomycin administration caused a significant increase in airway stiffness, as judged by the elevation of PAO in the fibrotic positive controls (Bleomycin+Vehicle) compared with the non-fibrotic negative ones (Naïve), both in WT and PARP-1^−/−^ mice (Figure 1).

After 21 days, the treatment with JNJ significantly reduces PAO both in WT and in PARP-1^−/−^ mice, in comparison to the relative group of animals treated with Vehicle; while VUF treatment seems to be not significantly effective in reducing the bleomycin-induced airway stiffness (Figure 1). The selected doses were based on previous papers (Pini et al., 2012; Lucarini et al., 2016). These results confirm our previous observation that H_4_R ligands could have beneficial effects in decreasing lung fibrosis in WT (Lucarini et al., 2016) and in PARP-1^−/−^ animals, indicating that the treatment with H_4_R antagonists exerts its beneficial effects also in situation of PARP-1 deletion.

### Changes in PARP-1 Activity

It has been demonstrated that bleomycin administration increases PARP activity in mouse lung tissues (Genovese et al., 2005). Moreover, previous studies provided evidence that PARylated protein levels, the major products of PARP activity, are significantly increased in lung homogenates of WT mice treated with bleomycin, while the treatment with HYDAMTIQ, a potent PARP-1/2 inhibitor, dose-dependently, prevents this PARylation (Lucarini et al., 2017).

**Figure 2:**
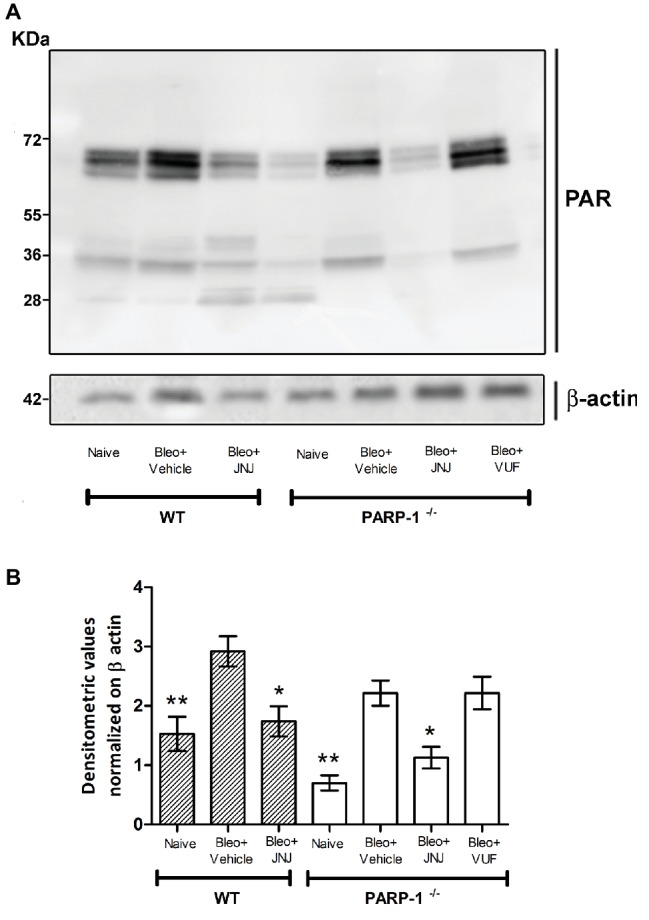
PARP activity. **(A)** Western blot analysis of PARylated protein content in lung samples from each experimental group. **(B)** Densitometric analysis was normalized on β-actin (*n* = 10 animals per group). *p < 0.05 and **p < 0.01 *vs.* Bleo+Vehicle of each related group WT or PARP^−/−^ (Bleo = Bleomycin).

To evaluate the effects of intra-tracheal administration of bleomycin on PARP activity, we investigated the formation of polyADP ribose (PAR) polymers in mouse lung tissues in WT and PARP-1^−/−^ mice. As shown in Figure 2 and in Supplementary Figure 1, Western blot analysis with anti-PAR antibodies report an increase in PARylated protein content in lung homogenates of WT and in PARP-1^−/−^ mice treated with bleomycin (Vehicle), compared to non-fibrotic controls (Naїve). Treatment with the H_4_R antagonist reduces the PARylated protein content in WT animals. Interestingly, our results indicate a reduction of PARylated protein levels in the lung tissues of PARP-1^−/−^ mice treated with JNJ, compared to those treated with VUF, suggesting a positive role of H_4_R antagonism in suppressing catalytic activity of PARP enzymes different from PARP-1.

### Histological and Morphometric Analysis

**Figure 3:**
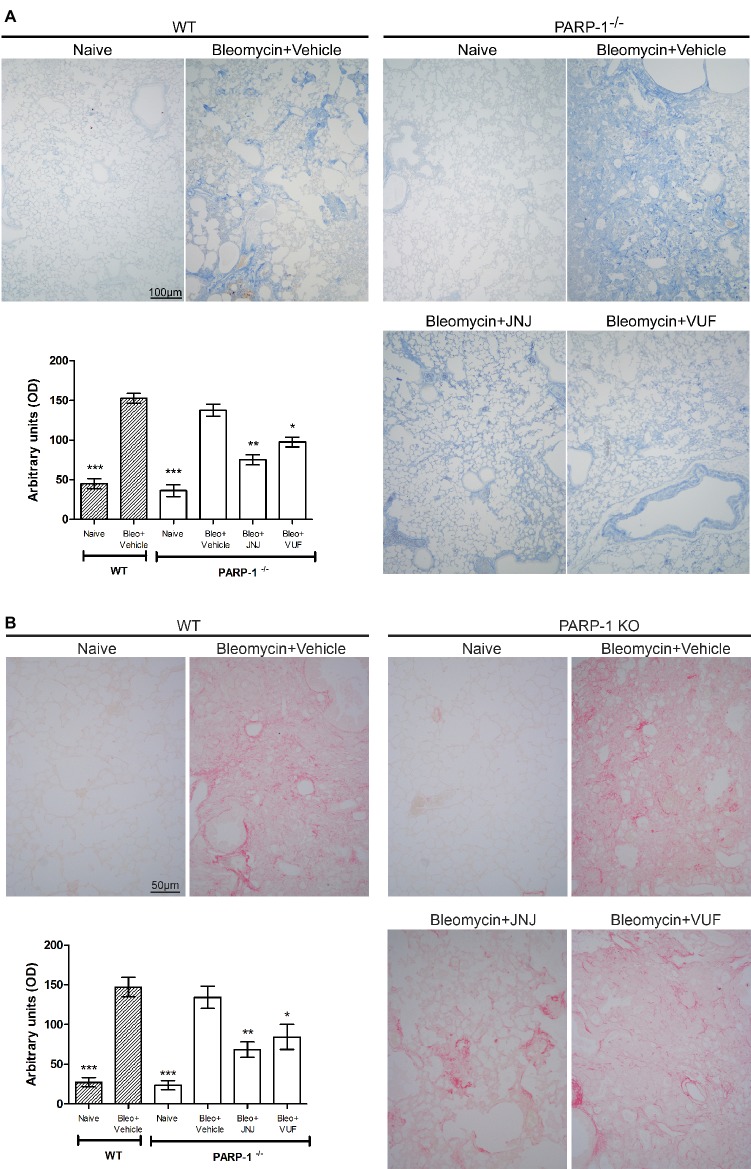
Evaluation of lung fibrosis. **(A)** Representative micrographs of Azan-stained sections from mice of the different experimental groups. Collagen fibers are stained deep blue. The lung from fibrotic controls treated with vehicle show marked fibrosis in peribronchial stroma, which is absent in non-fibrotic negative control lungs (Naïve) and reduced in JNJ treated animals. Bar graph showing the optical density (OD) (means ± SEM) of Azan-stained collagen fibers of the different experimental groups (*n* = 10 animals per group). *p < 0.05, **p < 0.01, and ***p < 0.001 *vs.* Bleomycin+Vehicle of each related group. **(B)** Representative micrographs of Picrosirius red-stained sections from mice of the different experimental groups. Collagen fibers are stained in red. Bar graph showing the optical density (OD) (means ± SEM) of Picrosirius red-stained collagen fibers of the different experimental groups (*n* = 10 animals per group). *p < 0.05, **p < 0.01, and ***p < 0.001 *vs*. Bleomycin+Vehicle of each related group.

Acute inflammatory events and deposition of collagen fibers cause a pathological remodeling of the parenchyma of the lungs. Morphological observation and computer-aided densitometry on Azan-stained sections (Figure 3A) reveal a significant increase in collagen deposition in the lungs of all bleomycin-treated animals (Vehicle) compared to the non-fibrotic negative controls (Naïve). As already published, in WT animals, H_4_R antagonists reduce the deposition of collagen fibers in lungs compared to Vehicle (Rosa et al., 2014; Lucarini et al., 2016). The treatment with JNJ causes an important and significant reduction of the amount of lung collagen fibers in PARP-1^−/−^ mice compared to Vehicle group. A slight effect is reported in VUF treated mice. To confirm the assessment of lung collagen, the Picrosirius red was used as second staining method; this specific staining for collagen (Lattouf et al., 2014; Marcos-Garcés et al., 2017) confirms the results obtained with Azan staining (Figure 3B).

**Figure 4:**
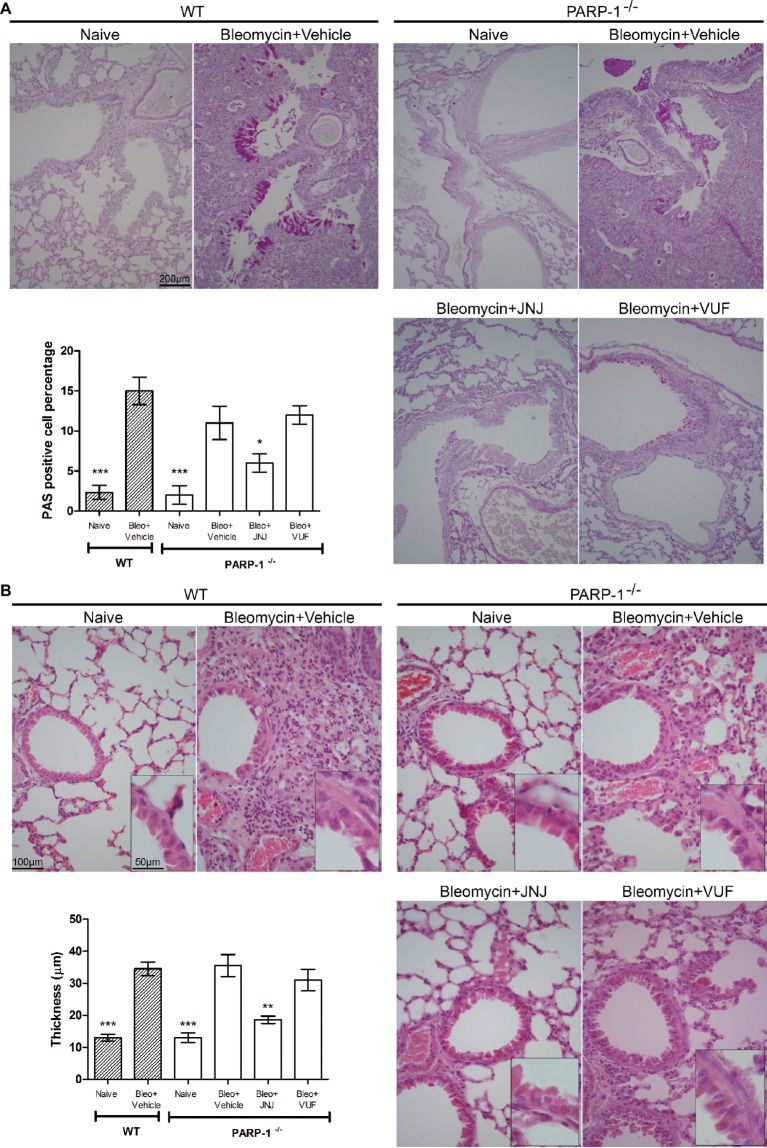
**(A)** Goblet cell hyperplasia. Representative micrographs of PAS-stained sections. Bar graph showing fraction of goblet cells (% means ± SEM) in the different experimental groups (*n* = 10 animals per group). *p < 0.05 and ***p < 0.001 of *vs.* Bleo+Vehicle of each related group. **(B)** Evaluation of muscular remodeling. Smooth muscle thickness was assessed by computer aided morphometry on H&E-stained lung sections. Representative micrographs of the sections. Bar graph showing the thickness of the muscular fiber (means ± SEM) in the different experimental groups (*n* = 10 animals per group). **p < 0.01 and ***p < 0.001 *vs.* Bleo+Vehicle of each related group.

We also evaluated the bronchial remodeling by measuring the number of goblet cells and the thickness of the smooth muscle layer, key histological parameters of inflammation-induced adverse airway remodeling (Bai and Knight, 2005). These two parameters are increased after intra-tracheal injection of bleomycin, both in WT and PARP-1 deficient mice. The treatment with JNJ significantly reduces the percentage of PAS-positive goblet cells over bronchial epithelial cells (Figure 4A), as well as the thickness of the airway smooth muscle layer (Figure 4B). On the contrary, VUF administration has no effect on both histological parameters, confirming our previous published results that on the effect of H_4_R antagonists on goblet cells and thickness of the smooth muscle layer in lungs, (Rosa et al., 2014; Lucarini et al., 2016).

### TGF-β Signaling Pathway

**Figure 5:**
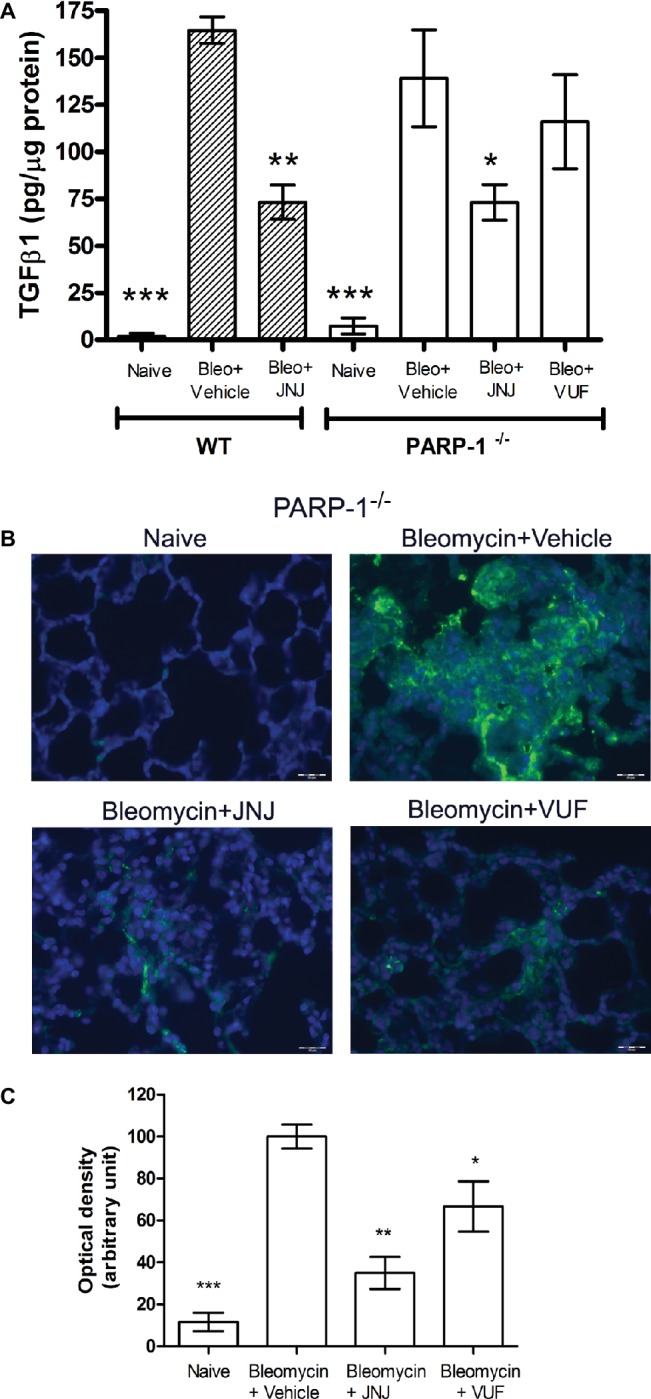
**(A)** Evaluation of TGF-β1 production. Bar graph showing lung tissue levels of the profibrotic cytokine TGFβ1 (means ± SEM) of the different experimental groups (*n* = 10 animals per group). *p < 0.05, **p < 0.01, and ***p < 0.001 *vs.* Bleo+Vehicle of each related group. **(B)** Evaluation of fibroblast activation. Immunofluorescence staining of lung tissue sections labeled with alpha smooth muscle actin (αSMA) (green) and nuclei (blue) counterstained with DAPI (20×). Images in the panels show the inhibition of αSMA expression, a marker of the transformation of fibroblasts into myofibroblasts (*n* = 10 animals/group). **(C)** Bar graph shows the optical density (means ± SEM) in the different experimental groups (*n* = 10 animals per group). *p < 0.05 and **p < 0.01 *vs.* Bleo+Vehicle. (Bleo = Bleomycin).

An increased TGF-β expression and an amplified TGF-β signaling through Smad pathways contribute to the establishment and development of pulmonary fibrosis (Leask and Abraham, 2004; Chen et al., 2013; Chitra et al., 2015). Our results clearly indicate that TGF-β, a major pro-fibrotic cytokine, is increased in the vehicle groups, both in WT and PARP-1^−/−^ mice. Systemic administration of JNJ causes a significant decrease of the levels of TGF-β in WT as well as PARP-1^−/−^ animals, while the treatment with the H_4_R agonist has no effect on the production of this cytokine (Figure 5A). These results demonstrate the anti-fibrotic activity of JNJ both in WT and in PARP-1^−/−^ mice.

### Fibroblast Activation

Transforming growth factor-β signaling has been reported to regulate the expression of αSMA, a marker of fibroblast activation, and myofibroblasts differentiation (Martin et al., 2007; Conte et al., 2014). To investigate the expression of αSMA during the fibrotic process, we performed immunofluorescence analysis in mouse lung tissues. Our results show a significant increase of αSMA levels in bleomycin-exposed PARP-1^−/−^ animals (Figures 5B,C). Treatment with JNJ significantly reduces αSMA levels in lung tissue of PARP-1^−/−^ mice compared to the vehicle group. Systemic administration of the H_4_R agonist provides no changes in αSMA lung levels. These results indicate that the use of an H_4_R antagonist, in the absence of PARP-1 enzyme, reduces the activation of fibroblasts and myofibroblasts differentiation and consequently the development of progressive fibrotic disease in bleomycin-exposed animals.

### Effects of H_4_R Ligands on Pro-Inflammatory Cytokine Production

**Figure 6:**
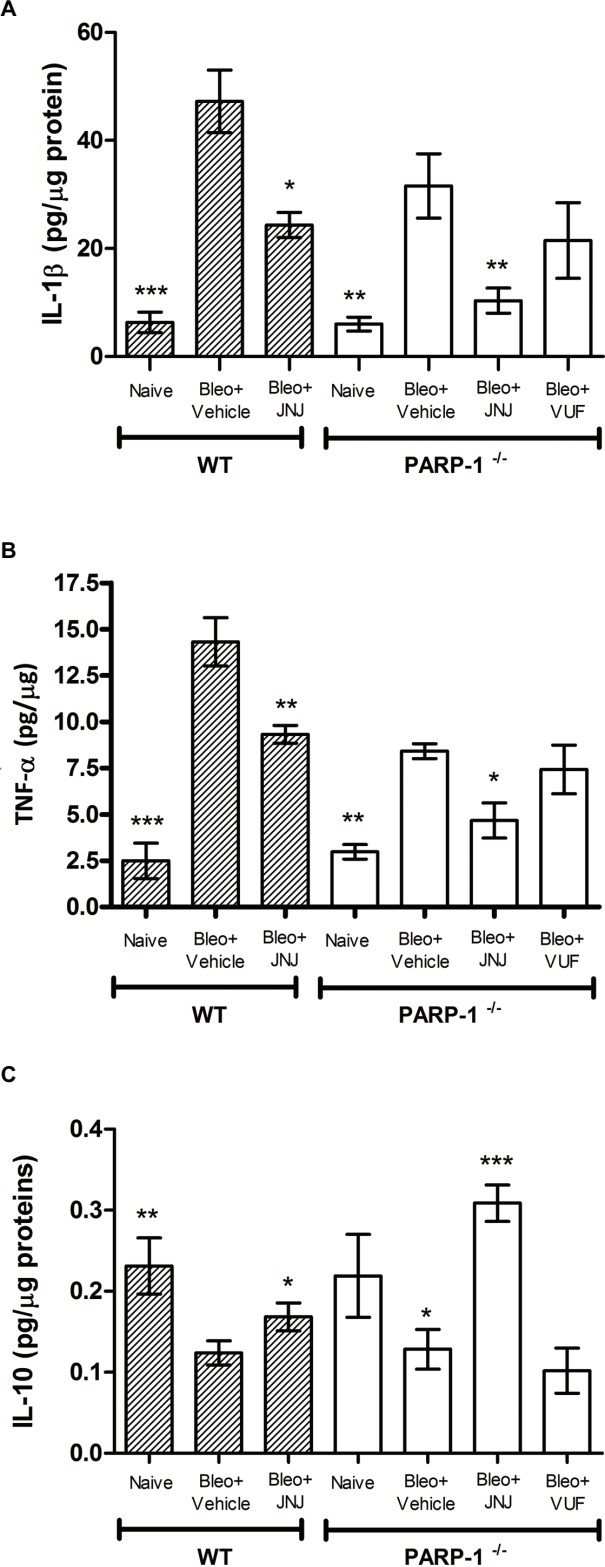
Determination of IL-1β **(A)**, TNF-α **(B)**, and IL-10 **(C)** levels in the supernatant of lung tissue homogenates. The values are expressed as pg/μg of total proteins (*n* = 10 animals per group). Values are mean ± SEM. *p < 0.05, **p < 0.01, and ***p < 0.001 *vs.* Bleo+Vehicle of each related group (Bleo = Bleomycin).

Chronic inflammation is determined by pathological wound-healing response, resulting in the accumulation of permanent scar tissue at the site of injury and consequently in the development of progressive fibrotic disease (Wynn and Ramalingam, 2012). Based on this evidence, we evaluated the late (21 days after challenge) inflammatory response to bleomycin by measuring the pro-inflammatory cytokines IL-1β and TNF-α, in lung homogenates of WT and PARP-1^−/−^ mice. Bleomycin treatment increases the levels of IL-1β and TNF-α both in WT and PARP-1^−/−^ mice; of note, in PARP-1^−/−^ mice, this increase is less pronounced in comparison to WT mice. The systemic administration of JNJ significantly reduces the increase in both groups of animals, but in PARP-1^−/−^ mice, this reduction is more evident. The treatment with VUF is not effective in the reduction of inflammatory cytokine production (Figures 6A,B).

In order to confirm the anti-inflammatory activity of the H_4_R antagonist, we evaluated the levels of interleukin-10 (IL-10), the most potent anti-inflammatory cytokine involved in resolution of different acute and chronic inflammatory diseases (Ajuebor et al., 1999). Our results report a significant increase in the production of IL-10 in WT and PARP-1^−/−^ mice treated with JNJ in comparison to PARP-1^−/−^ mice treated with vehicle or VUF (Figure 6C).

Our findings support the hypothesis that H_4_R antagonism exerts anti-inflammatory and anti-fibrotic effects in a model of bleomycin-induced lung fibrosis.

### Determination of Leukocyte Lung Infiltration and of Oxidative Stress Marker

**Figure 7:**
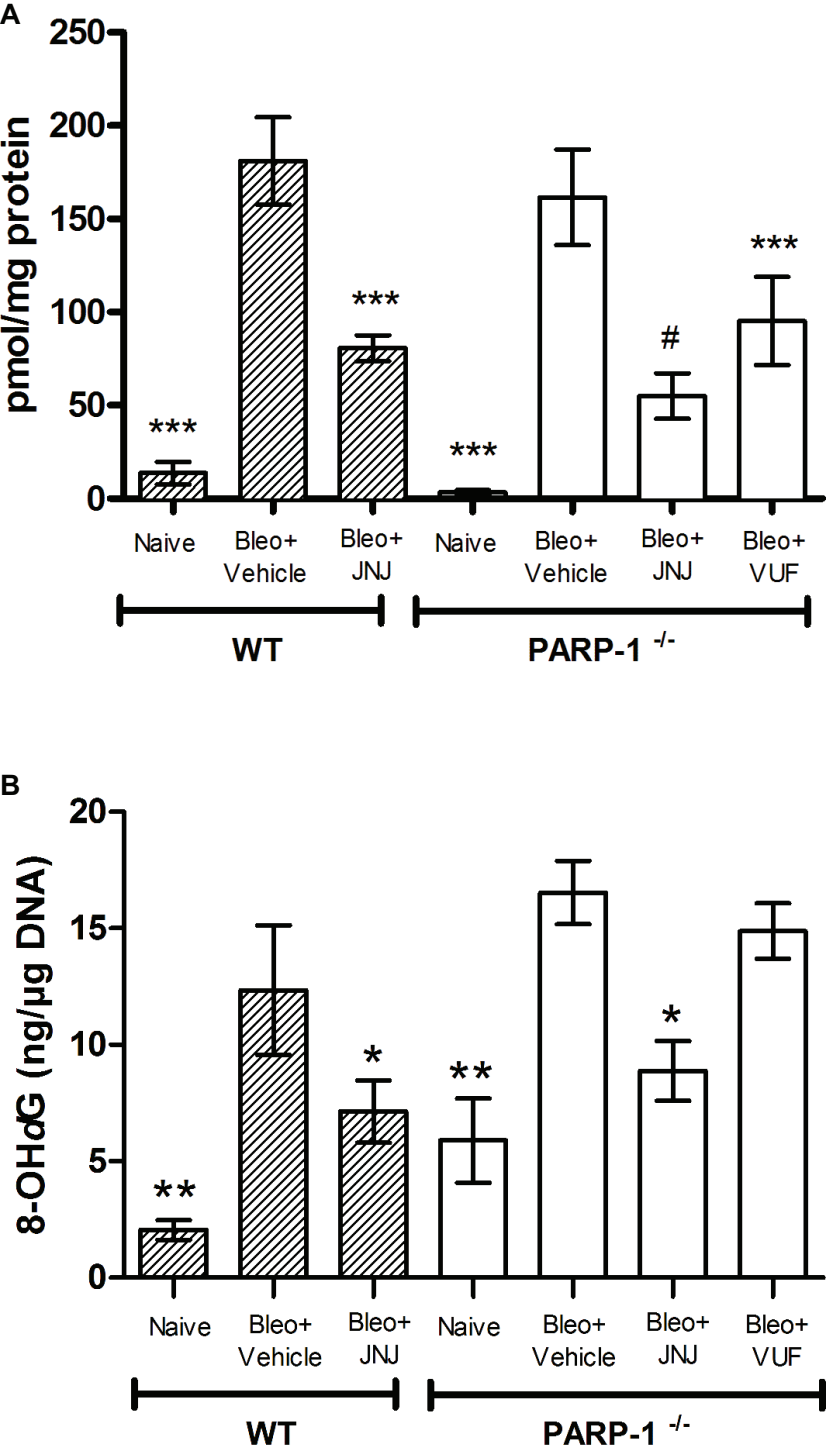
**(A)** MPO levels, a marker for leukocyte infiltration, are shown in the histogram. Values are expressed as mean ± SEM, expressed as picomoles per milligram of lung tissue protein (*n* = 10 animals per group) ***p < 0.001 *vs.* Bleo+Vehicle of each related group (Bleo = Bleomycin); #*p* < 0.05 *vs.* Bleo+VUF. **(B)** Evaluation of oxidative stress parameter in lung tissue. Bar graph shows the levels of 8-OHdG, a marker of free radicals-induced by DNA damage, (mean ± SEM) in the different experimental groups (*n* = 10 animals per group). *p < 0.05, **p < 0.01 *vs.* Bleo+Vehicle of each related group (Bleo = Bleomycin).

Lung MPO is a peroxidase enzyme abundantly expressed in neutrophils and monocytes/macrophages granules, and it is considered a reliable marker for leukocyte accumulation in inflamed tissues (Mullane et al., 1985). Levels of MPO were very low in the Naïve groups of both WT and PARP^−/−^ mice; MPO levels increased significantly in the bleomycin-treated mice (Vehicle groups of WT and PARP^−/−^ mice). A significant decrease in MPO was demonstrated after treatment with JNJ in WT and PARP^−/−^ groups of animals in comparison to Vehicle; treatment with VUF has some non-significant effects (Figure 7A).

The determination of 8-OHdG, a biological marker of DNA damage under oxidative stress, demonstrates that it is significantly increased in bleomycin-exposed animals (Vehicle), compared with non-fibrotic negative ones (Naïve). Interestingly, our results show a significant reduction of 8-OHdG levels in WT and PARP-1^−/−^ animals treated with JNJ (Figure 7B), but not with VUF.

## Discussion

Pulmonary fibrosis is an unmet medical need with a median survival of ~3 years since diagnosis. It is often severe and difficult to manage, resulting in a chronic condition that negatively affects the quality of life. The pharmacological therapy of pulmonary fibrosis is challenging, and for many patients, effective treatment is lacking. Therefore, novel therapeutic strategies are required (Wynn, 2007; Paz and Shoenfeld, 2010).

Recent studies demonstrated the involvement of PARP enzymes in modulating airway inflammation and fibrosis. Particularly, PARP-1 inhibition improves functional, biochemical, and morphometric parameters in an *in vivo* allergen-induced asthma-like reaction model (Lucarini et al., 2014) and in a bleomycin-induced pulmonary fibrosis model (Lucarini et al., 2017).

Histamine H_4_ receptor (H_4_R), the last discovered histamine receptor subtype, is functionally expressed and distributed in white blood cells, mast cells, eosinophils, dendritic cells, and T cells (Gutzmer et al., 2009). Recent evidence strongly suggests that H_4_R ligands might be exploited as potential therapeutics in modulating allergy, inflammation, autoimmune disorders, and possibly cancer. In an animal model of allergic airway inflammation, H_4_R-*knockout* mice present lower inflammation, reduced pulmonary infiltrate of lymphocytes and eosinophils, and an attenuated Th_2_ response (Dunford et al., 2006). Moreover, blocking H_4_R in a model of pulmonary fibrosis alleviates the inflammatory response, reducing COX-2 expression and activity, leukocyte infiltration, TGF-*β* production, and collagen deposition (Lucarini et al., 2016).

Although these findings suggest a promising therapeutic use for H_4_R antagonists in the modulation of chronic lung diseases, the biochemical basis underlying the protective and beneficial effects of these drugs are unknown and need to be further elucidated.

Here, we tested the possible role that PARP-1 may have in the mechanism of action of H_4_R antagonists. Briefly, we demonstrate that JNJ, a selective antagonist of the histamine H_4_R, exerts its anti-inflammatory and anti-fibrotic properties independently of PARP-1 signaling pathway, in an *in vivo* mouse model of bleomycin-induced lung fibrosis.

Chronic inflammation is determined by pathological wound-healing response, resulting in the accumulation of scar tissue and consequently in the development of progressive fibrotic disease (Wynn and Ramalingam, 2012). Our model of pulmonary fibrosis is well described and widely accepted and includes an acute inflammatory phase (7–9 days), followed by a chronic inflammatory infiltrate and fibrotic process in the next 2 weeks, with maximal responses at day 21 (Moore and Hogaboam, 2008).

Therefore, we evaluated the inflammatory response to bleomycin in lung homogenates of WT and PARP-1^−/−^ mice. The results show that the treatment with JNJ significantly reduces the production of IL-1β and TNF-α pro-inflammatory cytokines and the activity of MPO, as well as the levels of 8-OHdG, a reliable marker of oxidative stress, in WT and in PARP-1^−/−^ mice. Collectively, our results demonstrate that H_4_R antagonist’s anti-inflammatory and anti-fibrotic effects are not dependent on PARP-1 expression, thus adding further evidence on the role of H_4_R in controlling leukocyte trafficking and pro-inflammatory responses (Zampeli and Tiligada, 2009).

Previous data demonstrated a role for histamine and H_4_R in the production of TGF-β, the major pro-fibrotic cytokine, and in fibroblast activation (Cowden et al., 2010). The cytokine TGF-β has been proposed to play a key role in lung fibrosis (Tatler and Jenkins, 2012), and drugs able to control TGF-β expression and/or signaling seem to be active in reducing fibroblast activation and clinical progression of the disease (Bonniaud et al., 2005). The TGF-β overexpression has been repeatedly associated to lung fibrosis (Sime et al., 1997), and the administration of TGF-β neutralizing antibodies was able to prevent the disease.

Here, we report the positive effects of the H_4_R antagonist on the lung TGF-β pathway, showing that the treatment with JNJ significantly reduces TGF-β levels also in PARP-1^−/−^ mice. Thus, our results indicate that H_4_R antagonists can ameliorate lung fibrosis independently of PARP-1 expression. To further confirm the effects of the H_4_R antagonist JNJ, we performed experiments also with VUF8430, introduced as a selective H_4_R agonist. Surprisingly, in our model, VUF has some unexpected effects in reducing features of lung injury, in functional assay as well as α-SMA and collagen deposition. This discrepancy could be explained because this H_4_R agonist with minor modification on imidazole moiety has affinity also for H_3_R (Lim et al., 2009), thus reducing the endogenous release of histamine and H_4_R activation.

PARP-1 activity contributes to lung fibroblast activation and induces their proliferation with increased expression of αSMA, which plays a pivotal role in lung fibrosis (Hu et al., 2013). In order to confirm the above results, we demonstrate a significant reduction of αSMA levels in PARP-1^−/−^ mice treated with JNJ, suggesting that the use of an H_4_R antagonist, in the absence of PARP-1 enzyme, strongly reduces the activation of fibroblasts and the differentiation of myofibroblasts, consequently blocking the development of progressive fibrotic disease.

Overall, these findings suggest that PARylation is a key factor for the pathogenesis of pulmonary fibrosis and provide evidence that PARP-1 and H_4_R are independently involved in the signaling pathways activated during inflammatory and fibrotic processes. The association of PARP-1 deficiency and H_4_R antagonist treatment exerts a cross-talk response with anti-inflammatory and anti-fibrotic effects, decreasing bronchoconstriction and airway inflammation, as also shown by the reduction of the percentage number of goblet cells and the thickness of the smooth muscle layer, key parameters of inflammation-induced adverse airway remodeling.

Actually, the treatment of idiopathic pulmonary fibrosis is based on the use of either pirfenidone, a drug able to reduce the production of fibrogenic mediators, such as TGF-β, and inflammatory mediators, such as TNFα and IL-1β (Inomata et al., 2015), or nintedanib, a tyrosine kinase inhibitor able to reduce the transduction pathway of growth factor receptors, such as platelet-derived growth factor receptor, fibroblast growth factor receptor, and vascular endothelial growth factor receptor, and to reduce the transduction pathways leading to cell activation and proliferation (Nanthakumar et al., 2015). Both agents have recently been introduced into clinical practice showing their ability to ameliorate lung fibrotic processes; however, it is still not clear whether these agents have a clinically meaningful efficacy in long-term patient survival (Karimi-Shah and Chowdhury, 2015). Effective therapies to contrast airway inflammation and remodeling are not available, and novel therapeutic strategies are needed as alternative options when the standards of care are not enough.

In this study, we showed that the beneficial effects of H_4_R antagonists in reducing progressive pulmonary fibrosis are not dependent upon PARP-1.

In conclusion, the therapeutic potential of the combination of H_4_R antagonists with non-toxic doses of selective PARP-1 inhibitors could significantly reduce the development of pulmonary fibrosis. Although JNJ itself is emerging as a promising therapeutic agent in lung inflammation, the combination with PARP inhibitors could have an advantage over the single drug for the potentiating effect on the inhibition of inflammatory and pro-fibrotic parameters.

## Ethics Statement

The study protocol complied with the Declaration of Helsinki and the recommendations of the European Economic Community (86/609/CEE) on animal experimentation and was approved by the animal Ethical and Care Committee of the University of Florence (Florence, Italy) and by the Health Ministry (Authorization n 874/2017-PR). Experiments were carried out at the Centre for Laboratory Animal Housing and Experimentation (CeSAL), University of Florence. All studies involving animals are reported in accordance with the ARRIVE guidelines for reporting experiments involving animals (McGrath et al., 2010).

## Author Contributions

LL, MD, CL, and EM designed the research study. LL, MD, CL, SS, PN, and AP performed the research. AP and LL analyzed the data. LL, MD, and EM wrote the paper. FM revised the scientific content of the manuscript.

### Conflict of Interest Statement

The authors declare that the research was conducted in the absence of any commercial or financial relationships that could be construed as a potential conflict of interest.

## Footnotes

^1^http://rsb.info.nih.gov/ij

^2^http://rsbweb.nih.gov/ij

## Abbreviations

αSMA: Alpha-smooth muscle actin
ECM: Extracellular matrix
8-OHdG: 8-Hydroxy-deoxy-guanosine
H_4_R: Histamine H_4_ receptor
IL-10: Interleukin-10
IL-1β: Interleukin-1β
i.p.: Intra-peritoneal
IPF: Idiopathic pulmonary fibrosis
JNJ: JNJ7777120
MPO: Myeloperoxidase
OD: Optical density
PARP: Poly(ADP-ribose) polymerase
PAO: Pressure at the airway opening
PAS: Periodic acid-Schiff
PGE_2_: Prostaglandin E_2_
TGF-β: Transforming growth factor-β
TNF-α: Tumor necrosis factor-α
WT: Wild-type
VUF: VUF8430.
