# Supplementary figures and images for: Effects of PARP-1 Deficiency and Histamine H4 Receptor Inhibition in an Inflammatory Model of Lung Fibrosis in Mice
Source: Front Pharmacol. 2019 May 16;10:525. doi: 10.3389/fphar.2019.00525 (PMC6535496; doi:10.3389/fphar.2019.00525)

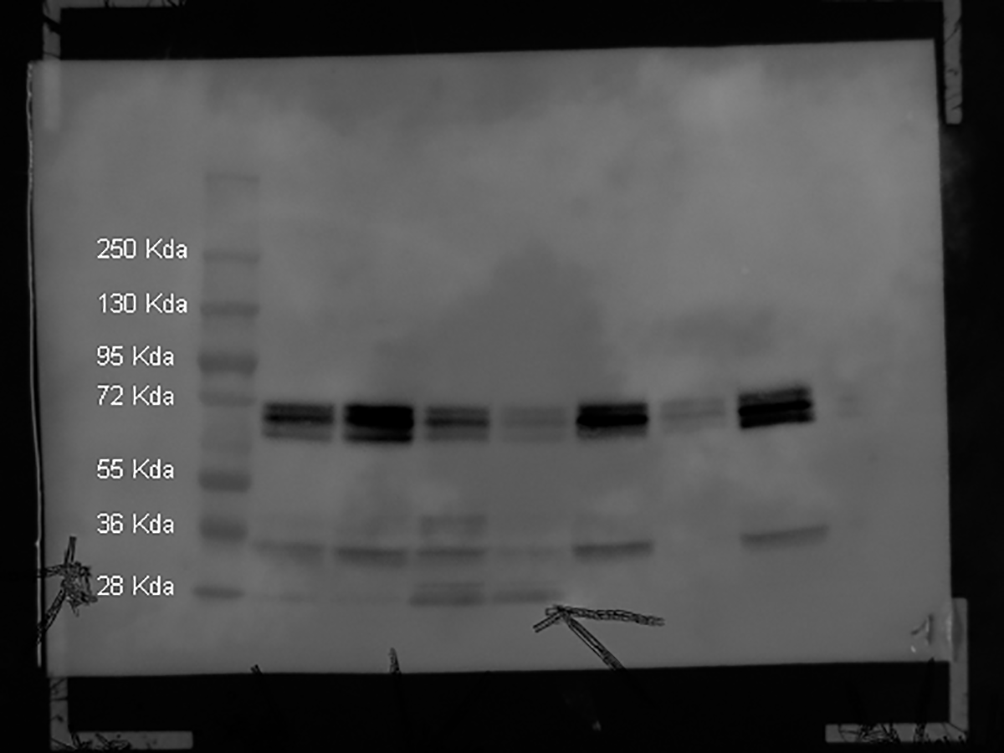

Supplement: Supplementary file 1 [file Image_1.tif]
